# Childhood sexual abuse and post-cesarean pain

**DOI:** 10.1002/pmf2.70178

**Published:** 2025-11-17

**Authors:** Jennifer L. Bailit, Trisha M. Boekhoudt, Kara M. Rood, Marcela C. Smid, Sindhu K. Srinivas, Hyagriv N. Simhan, Brian M. Casey, Monica Longo, Ruth Landau, Anna Bartholomew, Amber Sowles, Dwight J. Rouse, John M. Thorp, Uma Reddy, George R. Saade, Suneet P. Chauhan, William A. Grobman, Cora MacPherson, Eunice Kennedy Shriver National Institute of Child Health and Human Development Maternal-Fetal Medicine Units (MFMU) Network

**Affiliations:** 1 Department of Obstetrics and Gynecology, Case Western Reserve University, Cleveland, Ohio, USA; 2 Department of Obstetrics and Gynecology, George Washington University Biostatistics Center, Washington, District of Columbia, USA; 3 Department of Obstetrics and Gynecology, Ohio State University, Columbus, Ohio, USA; 4 Department of Obstetrics and Gynecology, University of Utah Health Sciences Center, Salt Lake City, Utah, USA; 5 Department of Obstetrics and Gynecology, University of Pennsylvania, Philadelphia, Pennsylvania, USA; 6 Department of Obstetrics and Gynecology, University of Pittsburgh, Pittsburgh, Pennsylvania, USA; 7 Department of Obstetrics and Gynecology, University of Alabama at Birmingham, Birmingham, Alabama, USA; 8 Department of Obstetrics and Gynecology, National Institute of Child Health and Human Development, Bethesda, Maryland, USA; 9 Department of Obstetrics and Gynecology, Columbia University, New York, New York, USA; 10 Department of Obstetrics and Gynecology, Brown University, Providence, Rhode Island, USA; 11 Department of Obstetrics and Gynecology, University of North Carolina at Chapel Hill, Chapel Hill, North Carolina, USA; 12 Department of Obstetrics and Gynecology, University of Texas Medical Branch, Galveston, Texas, USA; 13 Department of Obstetrics and Gynecology, University of Texas Health Science Center at Houston, Children’s Memorial Hermann Hospital, Houston, Texas, USA; 14 Department of Obstetrics and Gynecology, Northwestern University, Chicago, Illinois, USA

**Keywords:** cesarean, opioids, pain, sexual abuse

## Abstract

**Background:**

Sexual abuse before the age of 10 is reported by 2.7% of US women. Chronic pain has been linked to sexual abuse, but little is known about acute postoperative pain in those with a sexual abuse history and no history of chronic pain. We hypothesized that those who report prepubertal sexual abuse experience more pain at 7 days after hospital discharge from a cesarean delivery.

**Objective:**

To evaluate whether patients who report prepubertal sexual abuse experience more pain 7 days after hospital discharge from a cesarean delivery.

**Study Design:**

Secondary analysis of a multicenter randomized trial of individuals who underwent cesarean at 31 US hospitals (2020–2022). Participants were excluded if they had chronic pain or were missing sexual abuse data. The primary outcome was moderate-to-severe worst pain (≥4 on a scale from 0 to 10), as assessed by the Brief Pain Inventory (BPI), 7 days after discharge. Secondary outcomes included BPI at 2 weeks post-discharge and 6 weeks and 90 days postpartum, Pain Catastrophizing Scale (PCS) and Physical Function (PF) scores at 6 weeks, Milligram Morphine Equivalents (MME) use in 24 h before discharge, and opioid use measured at 90 days (number of prescriptions beyond discharge and total number of tablets taken after discharge). Maternal characteristics were compared between those with and without prepubertal sexual abuse. Multivariable modeling was performed adjusting for prepregnancy body mass index (BMI), family history of substance use disorders, and Edinburgh depression score ≥13 at randomization.

**Results:**

Of the 4881 participants included in the analysis, 5.3% reported prepubertal sexual abuse. They were significantly more likely to have a BMI ≥30 (54% vs. 44%), a family history of substance use disorders (58% vs. 24%), and an Edinburgh depression score ≥ 13 (9% vs. 4%) (all *p* < 0.05). Multivariable models showed that a BPI score ≥ 4 was significantly higher 7 days post-discharge (75% vs. 57%; adjusted relative risk [aRR], 1.3 [1.2, 1.4]) in those reporting prepubertal sexual abuse. BPI scores remained significantly higher through 6 weeks (2 weeks: 42% vs. 30%; aRR, 1.3 [1.1, 1.6]; 6 weeks: 14% vs. 10%; aRR, 1.5 [1.01, 2.1]; 90 days: 5% vs. 5%; aRR, 0.9 [0.4, 1.8]). PCS score ≥ 12 (15% vs. 12%) and PF score below average (64% vs. 56%) were not significantly different at 6 weeks. Opioid use in the 24 h before discharge (median MME 22.5% vs. 15, *p* < 0.01), additional opioid prescriptions after discharge (10% vs. 6%; aRR, 1.7 [1.1, 2.5]), and median total opioid tabs used after discharge (9% vs. 4 tabs, *p* < 0.01) were all significantly higher among individuals reporting prepubertal sexual abuse.

**Conclusion:**

In postpartum individuals who reported prepubertal sexual abuse, post-cesarean pain through 6 weeks and prescription opioid use were significantly higher. These associations warrant further study.

## 1 ∣ INTRODUCTION

Sexual abuse is common; an estimated 3.2 million women or 12.7% who report a history of sexual violence, report a history of first attempted or completed rape before the age of 10 [1].

Survivors of early childhood sexual abuse frequently experience long-lasting and life-long physical and mental health effects [2-6]. Sexual abuse has been associated with psychiatric diagnoses such as depression and anxiety [2, 5] and chronic pelvic pain [3, 4]. Childhood traumas, especially those occurring in early childhood, are associated with increased risk of depression and suicidal ideation later in life [6].

Although the association between childhood sexual abuse and chronic pain is well-established [3, 4], less is understood about the association of childhood sexual abuse and acute postoperative pain in the absence of a history of chronic pain. The objective of this study was to evaluate whether post-cesarean acute pain ratings were different between individuals with and without a history of childhood sexual abuse in a cohort without a history of chronic pain.

## 2 ∣ MATERIALS AND METHODS

This is a secondary analysis of a multicenter, non-inferiority, randomized controlled trial performed at the 31 centers participating in the *Eunice Kennedy Shriver* National Institute of Child Health and Human Development Maternal-Fetal Medicine Units Network from 2020 to 2022 [7]. In the parent trial, all postpartum individuals with a cesarean birth of a singleton, twin, or triplet pregnancy were screened for eligibility. Individuals were excluded if they had (1) undergone a combined vaginal–cesarean birth, (2) filled an antepartum opioid prescription, (3) a history of opioid use disorder, a contraindication to opioids or both acetaminophen and ibuprofen, (4) undergone significant surgical procedures (e.g., hysterectomy) during their cesarean birth, (5) a fetal or neonatal death prior to randomization, or did not speak English or Spanish or had previously participated in the trial. Individuals with cesarean deliveries were consented and randomized within 1 day before discharge either to a fixed number of opioids (20 tablets) or to a shared decision-making approach that determined the number of post-discharge opioid tablets. All patients with a cesarean delivery were potentially eligible for the trial, including patients with unplanned cesareans after spontaneous or induced labor and planned cesareans with no labor. Both general and neuroaxial anesthesia patients were eligible. For this analysis, participants were excluded if they had chronic pain or were missing sexual abuse data.

Baseline information was collected from medical record review and participant interviews at the time of study enrollment. The initial approach to the patient from research staff was on the discharge day or as close to discharge day as possible. Data collected included demographic information, the Opioid Risk Tool [8], the Edinburgh score for depression [9], and morphine milliequivalents (MME) use in 24 h before hospital discharge. Primary and secondary outcomes were the same as in the parent trial.

The primary exposure in our secondary analysis of history of preadolescent sexual abuse was derived from a question on the self-administered Opioid Risk Tool (ORT). The ORT questionnaire is a commonly used, validated tool designed to see who is at risk for opioid misuse before prescribing opiates [8]. Preadolescent sexual abuse question is a standard part of the tool as preadolescent sexual abuse has previously been tied to opioid abuse [10, 11]. The term preadolescence is not explicitly defined in the questionnaire, but for the purposes of the study was defined as less than 13 years of age. The ORT does not address any other forms of abuse such as verbal or physical. The ORT was collected after the patient delivered and was screened for the parent trial but before randomization in the 24 h before hospital discharge. After the patient filled out the ORT form, study personnel reviewed the sheet with the patient to go over any unclear answers. Data on other people in the room when the questionnaire was filled out were not collected and are unknown.

The primary outcome was moderate-to-severe worst pain (≥4 on a scale from 0 to 10) as assessed by the BPI, 1 week after discharge [12]. The primary outcome is the same as the parent trial and was chosen based on a six-center observational study in which the median pain score was 4 (interquartile range 3–5) at 1-week post-discharge [13]. Participant follow-up occurred at 2 weeks, 6 weeks, and 90 days after discharge by phone or in person. Secondary outcomes including BPI at 2 weeks post-discharge and 6 weeks and 90 days postpartum, Pain Catastrophizing Scale and Physical Function score at 6 weeks, and opioid use measured at 90 days (number of prescriptions beyond discharge and total number of tablets taken after discharge) were determined at the follow-up visits. Pill usage was assessed by patient report.

A priori, it was specified that covariates would be included in multivariate models if there were both a clinically significant imbalance across groups and there could be a plausible relationship to the study outcome. If clinically significant baseline imbalances were found in the variables chosen a priori, they were included in the models. Two variables were collected that evaluated a personal history of depression. The first was a question collected at study enrollment that asked about any history of depression or anxiety, the other was an Edinburgh Postpartum Depression Score (EPDS) score from study enrollment that reflected ongoing depression. EPDS, which measures current depression, was included in the model. Continuous variables were compared using the *t*-test or Wilcoxon rank-sum test and categorical variables using chi-square or Fisher’s exact test. Log-binomial regression was used to estimate adjusted relative risks (aRRs) and 95% confidence intervals (CIs) for categorical outcomes.

## 3 ∣ RESULTS

**FIGURE 1:**
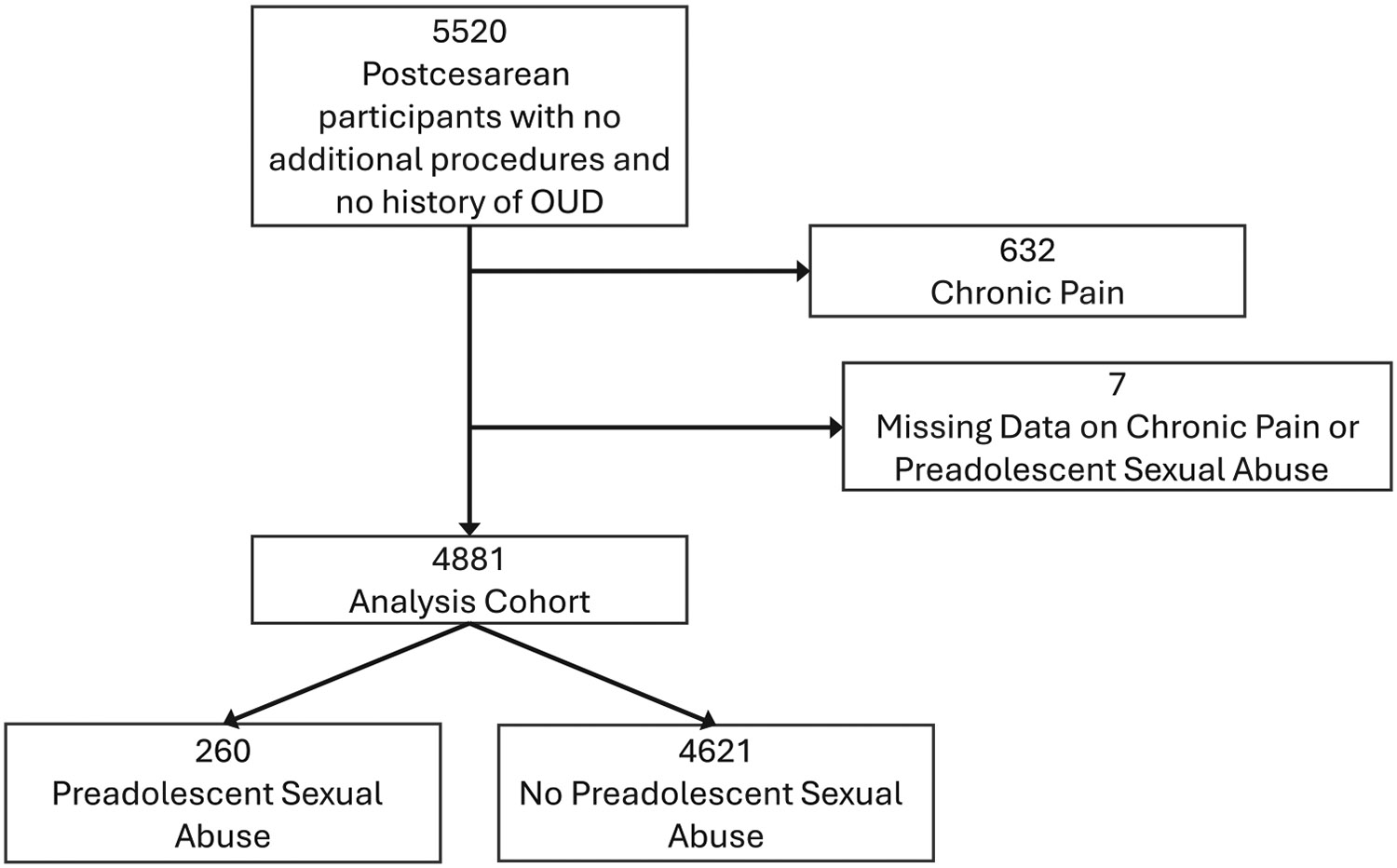
Flowchart of participants.

In the parent study, 5520 participants were enrolled. Of these, 632 were excluded from this analysis for chronic pain and 7 had missing data on chronic pain or preadolescent sexual abuse, leaving 4881 individuals for analysis (Figure 1). There were no differences in preadolescent sexual abuse by the parent study randomization group, nulliparity, or breastfeeding at 1 week (Table 1). There were small but statistically significant differences in preadolescent sexual abuse by age and race. Preadolescent sexual abuse was associated with higher body mass index (BMI) (10% more people with BMI ≥30), lower household income (10% more likely to have an income <$35,000/year), and more than double the proportion with depression, anxiety, and personal and family history of substance use disorder (Table 1).

At hospital discharge, the odds of having pain, rated as at least a 4 on the 10-point BPI scale, were similar between participants with and without preadolescent sexual abuse (aRR, 1.05; 95% CI, 1.0–1.11) (Table 2). However, at 1-week post-discharge, participants with a history of preadolescent sexual abuse had significantly increased risk of pain compared to those without a history (aRR, 1.28; 95% CI, 1.18–1.39). At both 2-week and 6-week postpartum, participants with a history of preadolescent sexual abuse were at increased risk of moderate-to-severe pain compared to those without this history (Table 2). At 90 days postpartum, there was no longer a difference in risk of moderate-to-severe pain between the groups.

Participants with a history of preadolescent sexual abuse had higher levels of MME used in the 24 h before hospital discharge (median 22.5 vs. 15.0 *p* < 0.001), higher risk of one or more prescriptions for opioids filled beyond the discharge prescription (aRR, 1.67; 95% CI, 1.11–2.50), and more opioid tablets used since discharge (median 9 vs. 4 *p* < 0.001) (Table 3). PCS scores and PF scores were not statistically different between the groups after adjusting for obesity, family history of substance use disorder, and baseline EPDS ≥ 13 (Table 4). We chose to use EPDS ≥ 13, reflecting ongoing depression, instead of personal history of depression/anxiety. Note that the relationship between previous abuse and post-cesarean pain did not differ by whether the participant reported a history of depression or anxiety. Due to the limited number of participants reporting a personal history of substance use disorder, family history of substance was included in multivariate analyses.

## 4 ∣ DISCUSSION

Individuals delivered by cesarean who reported preadolescent sexual abuse experience more pain severity between 1-and 6-week postpartum than individuals without a history of preadolescent sexual abuse. Differences in the pain scores between the groups resolve by 90 days postpartum. Additionally, individuals with a history of preadolescent sexual abuse use more opioids in the hospital and the first 6 weeks after discharge.

Previous studies suggest that, in an experimental setting, women with a history of sexual abuse have altered acute pain processing [14, 15]. Our data, which demonstrate a clinical corollary to the laboratory data, show that preadolescent sexual abuse is associated with more severe post-cesarean pain than that reported by individuals without a history of preadolescent sexual abuse.

The mechanism by which preadolescent sexual abuse is associated with acute pain after cesarean remains unclear. Childhood maltreatment—including physical, sexual, and verbal abuse—affects the survivor’s epigenetics, which in turn may alter physiologic responses to future stress [16-20]. Potential pathways by which these epigenetic changes have long-term effects have been explored in animal models of neglect [16, 21, 22]. Some have suggested that epigenetic changes are a protective adaptation to prepare offspring for adverse environments [21, 23]. Other studies demonstrate differences in brain structure noted on imaging, as well as in hormonal profiles among those with and without childhood abuse histories [24-26]. Thus, while some survivors of childhood abuse may not have chronic pain, their bodies may reveal their traumatic histories in times of stress [23].

Sexual abuse is also associated with psychiatric conditions such as depression and anxiety [2, 5], which in turn may be associated with altered pain [27, 28]. Nevertheless, in our study, even after controlling for depression and anxiety, a significant relationship between preadolescent sexual abuse and post-cesarean pain trajectories remained. Better understanding of the mechanisms of how preadolescent abuse is associated with acute pain responses in adulthood is critical to improving long-term outcomes for survivors.

If the pathways by which abuse influences future pain responses later in life can be elucidated, it opens the possibility of intervention to prevent these responses. Administration of morphine early in the traumatic injury timeline can help mitigate the development of post-traumatic stress disorder (PTSD) [29]. While much work remains to be done, there is promising work being done to develop therapeutic interventions that could prevent some of the lifelong sequelae of abuse [16, 30, 31].

One of the limitations of our study is that we did not record the timing, severity, or repetitive nature of the preadolescent sexual abuse, and therefore our investigation of the relationship between sexual abuse and post-cesarean pain is limited. While our study focuses on preadolescent sexual abuse, other types of childhood maltreatment may have similar epigenetic and physiologic consequences [17, 20]. Future research into the pathways by which sexual abuse is associated with the experience of pain and analgesic use after cesarean delivery and any associated biological changes is warranted. Furthermore, the effect of postadolescent sexual abuse on acute pain is unknown. There is some evidence that PTSD following adult rape is associated with epigenetic changes suggesting that the epigenetic effects of trauma may not be limited to childhood [19]. Lastly, while data that morphine may decrease PTSD among individuals who have suffered trauma are promising, the use of pharmacologic interventions to mitigate sequelae from sexual abuse has yet to be explored.

It is possible that the link between preadolescent sexual abuse and post-cesarean pain is not from epigenetic changes at all, but from other causes. The preadolescent sexual abuse group and the no abuse group differ in many aspects (Table 1). Additionally, it is possible that there are unmeasured factors that affect individual’s ability to control post-op pain at home that would not affect their pain in the hospital thus explaining why there were differences BPI after discharge.

A strength of our study was that participants self-identified as having had preadolescent sexual abuse and that all the outcome measures were directly collected by trained medical personnel through interviews after the cesarean delivery. This large cohort of individuals enrolled in over 13 centers nationwide ensures that our findings are generalizable.

Our study evaluated only preadolescent sexual abuse in relation to acute postsurgical pain and the findings cannot be applied to patients with chronic pain. Some participants in the study who did not identify as having preadolescent sexual abuse may have experienced postadolescent sexual abuse, which may or may not have a similar effect on pain outcomes. Additionally, we have no data on the timing, repetitiveness, and associated injuries among participants with self-identified preadolescent sexual abuse. The ORT is not an ideal questionnaire to assess childhood sexual abuse. As this was a secondary study with excellent data quality on post-cesarean pain, we took advantage of the self-reported data on preadolescent sexual abuse that were available. Future studies should use a validated tool designed to assess childhood and adult sexual abuse. Because of the limited data on preadolescent sexual abuse, we are unable to evaluate whether certain ages, type of abuse, or duration of abuse are more closely associated with changes in acute pain. As there were no biological specimens taken in the parent study, we cannot directly investigate epigenetic changes.

## 5 ∣ CONCLUSIONS

Participants with a history of preadolescent sexual abuse reported more severe acute pain after cesarean delivery and used a larger amount of systemic opioids in the hospital and up to 6 weeks after delivery compared to those without a history of preadolescent sexual abuse. Pain scores at 90 days were the same, however, suggesting that the differences in reported pain diminish after the 6-week assessment. Future research into the mechanisms by which preadolescent sexual abuse affects pain after cesarean delivery or might be associated with increased experience of pain (unrelated to childbirth) is critical to determine whether therapeutic interventions can mitigate the psychological and physical harms from early sexual abuse.

## Figures and Tables

**TABLE 1:** Maternal demographic and baseline characteristics by history of sexual abuse.^a^

Characteristic	History of sexual abuse (*N* = 260)	No history of sexual abuse (*N* = 4621)	*p* value
Randomized to individualized opioid-prescription protocol group	127 (48.9)	2304 (49.9)	0.75
Maternal age (years)	29.7 (6.2)	30.5 (5.7)	0.02
Race/ethnicity			
Hispanic	44 (16.9)	1012 (21.9)	0.02
Non-Hispanic Black	90 (34.6)	1221 (26.4)	
Non-Hispanic White	102 (39.2)	1975 (42.7)	
Other/not reported	24 (9.2)	413 (8.9)	
Prepregnancy body mass index (BMI)			
Mean	32.9 (10.2)	30.3 (8.3)	<0.001
BMI ≥ 30 kg/m^2^	117/216 (54.2)	1651/3770 (43.8)	0.003
Private insurance	116 (44.6)	2531 (54.8)	0.001
Highest level of education			<0.001
High school or less	84 (32.3)	1383 (30.0)	
Some college/trade school	112 (43.1)	1187 (25.7)	
Bachelor’s degree	31 (11.9)	1049 (22.7)	
Some graduate or professional school	33 (12.7)	998 (21.6)	
Employed	143 (55.0)	2880 (62.4)	0.02
Married	114 (43.9)	2605 (56.4)	<0.001
Annual household income			<0.001
Less than $35,000	99 (38.1)	1333 (28.9)	
$35,000–$74,999	63 (24.2)	941 (20.4)	
$75,000–$149,999	55 (21.2)	976 (21.1)	
$150,000 or more	13 (5.0)	705 (15.3)	
Prefer not to answer	30 (11.5)	663 (14.4)	
Nulliparous	106 (40.8)	1974 (42.7)	0.54
Breastfeeding at 1 week visit	203 (83.9)	3484 (81.5)	0.35
History of substance use disorder (other than opioid use disorder)	46 (17.7)	150 (3.3)	<0.001
Family history of substance use disorder	150 (57.7)	1115 (24.1)	<0.001
Depression	163 (62.7)	1059 (22.9)	<0.001
Anxiety	165 (63.5)	1355 (29.3)	<0.001
History of applying for disability insurance	10 (3.9)	78 (1.7)	0.01
Edinburgh Postpartum Depression Score ≥ 13 at baseline	23 (8.9)	204 (4.4)	0.001
NICU admission	73 (28.08	1247 (26.99)	0.70
Unplanned visit for wound infection through 6 weeks postpartum	5 (1.92)	80 (1.73)	0.81

a Values are *n* (%) or mean (SD).

Abbreviation: NICU, NeoNatal Intensive Care Unit.

**TABLE 2:** Brief Pain Inventory (BPI) scores over time by history of sexual abuse.

	History of sexual abuse (*N* = 260)	No history of sexual abuse (*N* = 4621)	Relative risk & 95% CI	Adjusted relative risk^a^ & 95% CI
BPI worst pain ≥ 4				
Delivery discharge	253 (97.3)	4097 (88.7)	1.10 [1.07, 1.12]	1.05 [1.00, 1.11]
1 week post-discharge	179 (74.6)	2439 (57.4)	1.30 [1.20, 1.40]	1.28 [1.18, 1.39]
2 weeks post-discharge	99 (41.6)	1233 (29.5)	1.41 [1.21, 1.65]	1.32 [1.10, 1.58]
6 weeks postpartum	32 (14.0)	422 (10.1)	1.39 [1.00, 1.94]	1.47 [1.01, 2.12]
90 days postpartum	11 (4.8)	209 (5.2)	0.93 [0.51, 1.68]	0.88 [0.44, 1.78]

Abbreviations: CI, confidence interval; EPDS, Edinburgh Postpartum Depression Score.

a Adjusted relative risk controlling for obesity, family history of substance use disorder, and baseline EPDS ≥ 13.

**TABLE 3:** Opioid use up to 90 days postpartum, by history of sexual abuse.

	History of sexual abuse (*N* = 260)	No history of sexual abuse (*N* = 4621)	Relative risk or median difference & 95% CI	Adjusted^a^ relative risk or median difference & 95% CI
Morphine milliequivalents used in 24 h prior to randomization—median (IQR)	22.5 (6.3–45.0)	15.0 (0–30)	7.5 [2.5, 12.5]	7.5 [2.7–12.3]
One or more opioid prescription filled beyond that prescribed at discharge, *n* (%)	26 (10.2)	277 (6.2)	1.66 [1.13, 2.43]	1.67 [1.11, 2.50]
Number of opioid tablets used since discharge—median (IQR)	9 (2–19)	4 (0–14)	5 [3.5, 6.5]	4 [2.0–6.0]

Abbreviations: CI, confidence interval; EPDS, Edinburgh Postpartum Depression Score; IQR, interquartile range.

a Adjusted relative risk controlling for obesity, family history of substance use disorder, and baseline EPDS ≥ 13.

**TABLE 4:** Pain Catastrophizing Scale score and Physical Function score at 6 weeks after cesarean delivery by history of preadolescent sexual abuse.

	History of sexual abuse (*N* = 260)	No history of sexual abuse (*N* = 4621)	Relative risk & 95% CI	Adjusted relative risk^a^ & 95% CI
Pain Catastrophizing Scale score				
Median (IQR)	4 (0–9)	3 (0–7)		
≥12	35 (15.3)	483 (11.5)	1.33 [0.97, 1.82]	1.13 [0.79, 1.62]
Physical Function score				
Median (IQR)	45.1 (41.4–59.0)	48.3 (42.6–59.0)		
<50, below average	148 (64.1)	2330 (55.5)	1.15 [1.04, 1.28]	1.09 [0.97, 1.22]

Abbreviations: CI, confidence interval; EPDS, Edinburgh Postpartum Depression Score; IQR, interquartile range.

a Adjusted relative risk controlling for obesity, family history of substance use disorder, and baseline EPDS ≥ 13.

## Data Availability

Data are available upon request.
